# Evaluating High Ambient Temperature Effects on Milk Production in Local Tunisian Goats: Toward Resilient Breeding Strategies for Arid Environments

**DOI:** 10.3390/ani16010061

**Published:** 2025-12-25

**Authors:** Ahlem Atoui, Sghaier Najari, Manuel Ramón, Clara Díaz, Mouldi Abdennebi, Maria-Jesús Carabaño

**Affiliations:** 1 Laboratory of Livestock and Wildlife, Institute of Arid Regions, Medenine 4100, Tunisia; 2 National Institute for Agricultural and Food Research and Technology, Departamento de Mejora Genética Animal, Ctra de la Coruña km 7.5, 28040 Madrid, Spain; manuel.ramon@inia.csic.es (M.R.);

**Keywords:** local goat, HT, milk production, heat tolerance

## Abstract

This study evaluates the impact of high ambient temperature (HT) on milk production in Tunisian local goats using both fixed and random regression models with quadratic and cubic Legendre polynomials. Daily minimum (Tmin), maximum (Tmax), and average (Tavg) temperatures were tested as heat load indicators, measured on the milking day and averaged over the 1–3 preceding days. The deviance information criterion (DIC) consistently showed that models including temperature effects provided a better fit than a baseline model without heat load. Cubic polynomials showed superior accuracy compared with quadratic models, even if the differences were relatively small. The best model was obtained with Tavg on the milking day, followed closely by Tmax averaged across one or two preceding days. The population response showed a thermoneutral plateau at lower temperatures, followed by declines beyond the HT thresholds. For Tmax, moderate and severe thresholds were detected at 20–23 °C and 25–27 °C, respectively, while for Tavg, thresholds occurred at 11–13 °C and 16–19 °C. Milk losses ranged from 22 to 85 g/°C depending on the temperature indicator, representing an average 4–5% decline in daily yield per degree above thermoneutrality. High variability in individual responses was observed. Some goats maintained stable production, while others showed steep declines under HT, with slope differences reaching over 150 g/°C. Correlations of milk yield across contrasting thermal environments were low, indicating that animal ranking changes with temperature. High-producing goats are more affected by heat, showing the need for a balance between production and heat tolerance.

## Simple Summary

In the arid south of Tunisia, local goats are known for their ability to adapt and stay productive under harsh conditions. However, increasing temperatures intensified by climate change can reduce their milk production. In this study, the effect of high ambient temperature (HT) on milk yield was quantified in Tunisian local goats, considering milk production as a primary economic and nutritional trait in smallholder and pastoral systems. The results showed a significant negative effect of high ambient temperature with relevant reductions in milk yield under both moderate and severe heat load conditions. These findings provide valuable insights for designing selective breeding programs aimed at improving heat tolerance in dairy goats, leading to support the sustainability and productivity of goat farming in arid regions.

## 1. Introduction

Livestock production in arid and semi-arid regions is affected by climate change, especially by increasing temperatures and prolonged periods of heat. These climatic disruptions pose a serious threat to sustainable livestock systems and food security in arid environments. In the arid south of Tunisia, the climate is extremely dry, with annual rainfall often below 200 mm and summer temperatures that can exceed 45 °C [1]. These harsh conditions create serious challenges for animal production, including reduced feed availability, limited water resources, and increased HT.

Despite the rich literature on dairy cattle, fewer studies have investigated the effects of HT in small ruminants, particularly goats. However, goats play an essential role in rural livelihoods in dry areas, contributing to climate resilient pastoralism due to their strong ability to survive and produce under difficult conditions [2,3]. Goats possess specialized thermoregulatory mechanisms such as increased respiration rate, sweating, and behavioral adaptations (shade seeking, reduced activity) that help maintain homeostasis under HT [3]. Local goat breeds in Tunisia are known for their good adaptation to heat and water scarcity. Their small body size, reduced feed and water requirements, and efficient digestive system allow them to tolerate harsh conditions more effectively than sheep or cattle in arid environments [4]. Traits such as low metabolic rate and efficient nutrient utilization further contribute to their heat tolerance.

These goats are mostly raised in semi-extensive systems and have not been selected for high production but rather for survival and reproduction in difficult environmental conditions [1]. The Temperature-Humidity Index (THI) is widely used to quantify heat stress in goats, with moderate to severe stress typically occurring at THI values between 71 and 86 [4].

With increasing demand for goat milk, there is a need to improve the productivity of local goats. One option to improve milk yield is crossbreeding with exotic high producing breeds. This strategy could lead to the loss of valuable local genetic resources. Another option is to improve productivity through selection within the local population. While more sustainable, this approach could still reduce the ability of animals to tolerate environmental stress if adaptation traits are not carefully preserved during selection.

This highlights the need for breeding strategies that enhance milk performance while maintaining resilience to climatic extremes. Recent studies on the same population have mentioned how kid growth is affected by heat and have proposed methods to identify animals that grow better under high temperatures [1]. However, the impact of HT on milk production, considering both population-level and individual-level responses, remains poorly documented in Tunisian local goats and in goat populations in general. To our knowledge, the only studies dealing with individual response in production to heat load increases in local goats are those reported by Menéndez et al. [5] and Menéndez et al. [6], dealing with local specialized dairy breeds under intensive or semi-intensive systems of production. On the other hand, the target population in this study is a local population traditionally raised under a semi-extensive pastoral system with no productive specialization. In this context, the aim of this study is to evaluate the effect of HT on milk production in local goats raised in the arid regions of southern Tunisia. We focus on animal-by-environment interactions under climate stress to identify phenotypic plasticity and resilience, and we quantify both average and individual differences in milk yield response to high temperature. Understanding these physiological and phenotypic responses will help design future selection programs that improve milk production in climate-resilient livestock systems without losing natural adaptation to arid conditions.

## 2. Material and Methods

### 2.1. Experimental Site and Ethical Compliance

The animals studied are part of the experimental goat herd El-GORDHAB experimental goat herd, located in the Tataouine region of Tatouine- Tunisia (33°30′ N and 10°40′ E). Situated in southeastern Tunisia, between the Matmata Mountains and the Mediterranean Sea, this region experiences an arid continental Mediterranean climate. It is characterized by irregular precipitation, averaging around 200 mm annually. Summer, known for its scorching temperatures, is the hottest and driest season, with maximum temperatures reaching up to 47 °C.

All animal procedures followed routine herd management practices and complied with the European Directive 2010/63/EU for the protection of animals used for scientific purposes [7]. As no invasive procedures were performed, formal ethical approval was not required.

### 2.2. Herd Management

The local caprine population is characterized by its small size, with adult males averaging 73 cm in height and females averaging 60 cm. The average weight of adult males is approximately 35 kg, whereas females typically weigh around 24 kg [8].

The animals were managed in semi-intensive production system, with natural pasture serving as the principal feed resource. Both the availability and nutritional quality of the pasture show considerable variation throughout the year and between years. The main breeding season for the local goats takes place from June to August. The kidding season ranges from October to February, with the majority of births occurring in November and December. Female kids were first mated between 12 and 18 months of age, depending on their birth season and body condition. Goats received daily concentrate amounts adjusted for their body weight and lactation stage. Fresh water and mineral salt blocks were available at all times. Animals were vaccinated against enterotoxaemia and regularly treated for parasites.

Additionally, reproductive information relevant to the statistical analyses was recorded for all does. The year of kidding was documented from 2019 to 2024. The age of dam at kidding was classified into six categories (2 to 6 years old). Litter size was also registered at birth and categorized as either single or twin. These variables were included as fixed effects in all statistical models)

Milk records used in this study were collected from 2019 to 2025. Milk yield was recorded every two weeks from 7 days post-partum until 180 days of lactation. Milking was performed mechanically in the morning using a portable milking machine, after kids had been separated from their dams for approximately 12 h and reunited immediately after milking. Prior to milking, teats were disinfected with an iodine solution and dried with paper towels after about 30 s. Milk was collected in a graduated glass cylinder, and the volume was read at the liquid surface level. Because only one milking was performed per recording day, the daily milk yield was mimicked by multiplying the measured morning yield by two.

Daily maximum (Tmax), minimum (Tmin), and average (Tavg) temperatures were obtained for the milk recording period from a weather station from the national meteorological agency located 8 km apart from the farm. Temperatures were preferred over a temperature-humidity index due to the scarcity of humidity measurements recorded in the weather station. Moreover, the present study was conducted in a Mediterranean arid environment, where relative humidity remains consistently low throughout the year, particularly during the warm season. As a result, temperature is the dominant driver of heat load, while the contribution of humidity to the overall thermal stress is minimal [9]. Milk records were merged with temperature data by date of recording. For each date of milk recording, temperatures on the milking day (0) and average temperatures for a window period comprising the 1, 2, and 3 days previous to the date of milking were calculated. After edits and merging the data to remove missing or abnormal records, a total of 2281 milk production from 150 local goats were included in the study.

### 2.3. Statistical Analyses

#### 2.3.1. Models

Mixed models were applied to estimate both the average population response in daily milk to variations in heat load and individual deviations. Heat load was defined by temperatures on the milking day (Tmax_0_, Tmin_0_, Tavg_0_) and also considering the average of daily temperatures over the 0 and 1, 2, and 3 days preceding milking day (${\bar{T}}_{max0-1,2,3\;}{\bar{T}}_{min0-1,2,3\;}{\bar{T}}_{avg0-1,2,3\;}$).

Three groups of models of increasing complexity were considered: (i) a baseline model with no temperature effect; (ii) a population model including polynomial functions to describe the average population response to combinations of temperature type (Tavg; Tmax; Tmin) and period (day 0; days 0 to 1; days 0 to 2; and days 0 to 3 days before test day control); and (iii), finally, a model adding the random deviations of individual reactions to temperatures.

#### 2.3.2. Baseline Model (M_0_)

(1) $$y_{\mathrm{ijkl}}=Y_{i}+{\mathrm{DA}}_{j}+{\mathrm{LS}}_{k}+\sum_{k=0}^{k=2}\beta_{k}d_{kl}+e_{\mathrm{ijkl}}$$

where y_ijkl_ is the daily milk yield obtained from milking the goat, Y_i_ is the ith class of year of kidding (i = 2019, 2020, …, 2024) DA_j_ is the class of goat’s age at kidding (j = 2, 3, …, 6), LS_k_ is the class of litter size for the corresponding kidding (k = simple, double), $\sum_{k=1}^{2}\beta_{k}d_{kl}$ represents the expected milk yield at day l of lactation using a quadratic polynomial to describe the expected lactation curve, with $\beta_{k}$ being the kth regression coefficient and d_kl_ the kth covariable for the lth day in milk, and e_ijkl_ is the residual term, assumed homogeneous and independent across observations, i.e., var(e) = $I\sigma_{e}^{2}$.

#### 2.3.3. Average Population Response to Temperature Increase Models (M_FR_)

(2) $$y_{\mathrm{ijklt}}={\mathrm{SEF}}_{\mathrm{ijkl}}+\sum_{r=1}^{r=2,3}b_{r}x_{rt}+e_{\mathrm{ijklt}}$$

where y_ijklt_ and e_ijklt_ are the same as in Equation (1), SEF_ijkl_ = Y_i_ + DA_j_ + LS_k_ + $\sum_{k=0}^{2}\beta_{k}d_{kl}$ (as in Equation (1), for M_0_) represents systematic environmental factors influencing goat milk yield that are unrelated to heat load (i.e., temperature) and $\sum_{r=1}^{r=2,3}b_{r}x_{rt}$ is the expected response in milk yield at a given value t of heat load (temperature-period combination) provided by a quadratic or cubic Legendre polynomial function, with regressions b_j_, and x_rt_ the corresponding covariables for a given value of heat load T = t.

The average (population-level) response of milk goat at a given temperature was obtained from solutions for the regression coefficients (b_r_) in Equation (2), using the expression:

(3) $$E\left(y_{t}\right)=\sum_{r=1}^{r=2,3}\hat{b_{r}}x_{rt}$$

Evaluated along the range of observed values of each temperature, with x_rt_ being the Legendre polynomial covariates for temperature T = t. Quadratic (r = 0, …, 2) and cubic (r = 0, …, 3) polynomial functions for each temperature-period combination were analyzed.

The key parameters used to characterize the response to HT were the threshold temperatures at which high ambient temperature effects began and the associated milk losses. Thresholds were identified as change points in the polynomial response curve Equation (3), while milk losses were estimated from the slopes between those points. The method relies in a linearized version of the response curve by segmenting it in linear functions linked by changing or break points. Break points are identified when a change in the slope of response is detected. References for the statistical procedures used by the segmented package can be found in the segmented package web page (https://CRAN.R-project.org/package=segmented, accessed on 10 December 2025). In our case, two change points, that were supposed to identify mild and more intense HT, were searched.

#### 2.3.4. Individual Reaction to Temperature Models (M_RR_)

(4) $$y_{\mathrm{ijklnt}}={\mathrm{SEF}}_{\mathrm{ijkl}}+\sum_{r=1}^{r=2,}b_{rt}x_{rt}+\sum_{r=0}^{r=2}\alpha_{nr}x_{rt}+e_{ijklnt}$$

where y_ijklnt_, SEF_ijkl_, b_rt_, x_rt_ and e_ijklnt_ are the same as in Equation (2), and, $\alpha_{nr}$ are random regression coefficients describing the individual response for goat n. Quadratic polynomials were used to model both the overall and individual deviations.

For the random effects (the random regression coefficients, $\alpha_{nr}$, and the residual terms e_ijklnt_, the (co)variance structure was defined as:

$$var\left(\alpha_{n}\right)=G_{o}\left(\times \right)I,\;var\left(e\right)=I\sigma_{e}^{2}$$

where $\alpha_{n}$ is a vector containing the three random regression coefficients involving individual reactions to temperatures, the intercept ($\alpha_{0n}$), linear ($\alpha_{1n})$ and quadratic $(\alpha_{2n}$) coefficients for each goat n; G_o_ is a 3 × 3 matrix of (co)variances between random regression coefficients; I is an identity matrix with ones in the diagonal and zeroes otherwise, **e** is the vector of residual effects and $\sigma_{e}^{2}$ the homogeneous residual variance.

Individual deviations from the average response curve for each goat were obtained using the expression $\sum_{r=0}^{r=2}{\hat{\alpha }}_{nr}x_{rt}$, where ${\hat{\alpha }}_{nr}$ is the solution for the random regression coefficients in Equation (4) for goat n.

The key parameters at the individual level were: (i) the intercept, representing the goat’s level of milk at the average temperature (not varying with T), and (ii) the slope of milk change within the HT region, obtained as the derivative of the individual curve at a given value of the temperature. Because of the assumption of zero mean for random effects in mixed models, individual slope estimates reflect deviations from the population average. A positive slope indicates lower milk loss per unit increase in temperature compared with the population average, identifying the animal as more heat tolerant. Conversely, a strongly negative slope implies a faster decline in milk with increasing temperatures, indicating greater heat susceptibility. In matrix notation, slopes of the individual response curve of goat n at a given value of temperature, T, Slp_n_(T), were obtained as,

$${\mathrm{Slp}}_{n}(T)=C^{\prime }\alpha_{n}$$

and C′ is the matrix of first derivatives of the regression covariates with respect to temperature.

Using the estimated (co)variance components among random regression coefficients (G_o_), changes in milk variance across the temperature range and correlations between individual milk records at different temperatures were calculated as follows:

$$\mathrm{Var}(Z\alpha )=ZG_{o}Z^{\prime }$$

where Z is the matrix of covariates corresponding to the Legendre polynomial regressions (x_rt_) These estimates provide insight into the increase or decrease in trait variability under HT and the expected changes in animal ranking for milk across temperatures, reflecting differences in heat tolerance. 

Similarly, the (co)variability of individual response slopes to HT was derived from:

$$\mathrm{Var}\;(C^{\prime }\alpha )=C^{\prime }G_{o}C$$

Additionally, the covariance between intercepts (α_0_) and slopes of milk loss (Slp(T)) was estimated to assess the relationship between individual milk production (intercept) and heat tolerance (slope of milk loss under HT).

Overall, a total of 37 models were fitted, the baseline model, M_0_, 24 M_FR_ models to evaluate the effects of polynomial degree (quadratic vs. cubic) and the 12 combinations of temperature and period on the overall response (combinations of Tavg; Tmax; Tmin and four periods–day 0, 0–1, 0–2 and 0–3), and, 12 M_RR_ models including the 12 combinations of temperature period and using a quadratic polynomial to fit both population and individual reactions to temperatures. All models were estimated using the MCMCglmm package in R [10], based on a Bayesian framework. Each model ran for 10,000 iterations, with 2500 discarded as burn-in, being these parameters established by checking convergence of sampling chains. Posterior modes were used as parameter estimates. Models’ performance was compared using the Deviance Information Criterion (DIC), provided by MCMCglmm.

Quadratic and cubic polynomials were chosen to balance model flexibility and robustness: quadratic models are less sensitive to outliers, while cubic models capture more complex patterns when sufficient data are available. MCMC parameters were selected based on standard recommendations in the literature and convergence diagnostics, ensuring reliable posterior estimates. Farm-level microclimate variables were not recorded in this study; therefore, we used daily temperatures from the national meteorological agency as proxies.

## 3. Results

Table 1 details the summary statistics for milk production, days of lactation, and climatic indicators characterizing the environmental conditions to which the local goat population were exposed throughout the study period.

Milk production ranged from 0.20 to 5.20 kg, with 75% of goats producing more than 1.00 kg per day. The lactation period ranged from 7 to 180 days, with an average of 71 days, reflecting the wide variability in lactation stages represented in the data. The mean of maximum/minimum/average daily temperatures ranged from 14.12/0.03/6.5 °C to 43.6/24.74/33.29 °C. For all temperature types, mean values were similar across periods, but standard deviations decreased as the length of the period increased.

### 3.1. Average Population Response

**Figure 1:**
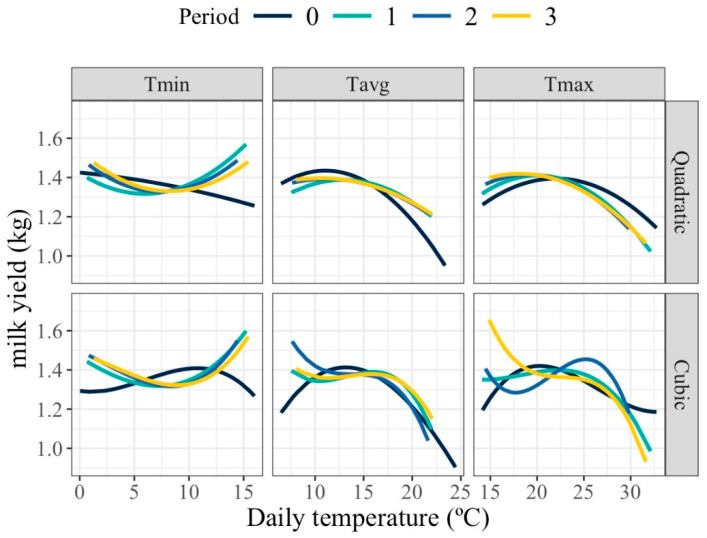
Expected average change in milk yield for different values of the average of minimum (Tmin), maximum (Tmax), and average (Tavg) daily temperatures of the day of milk recording (Period 0) or during that day and the previous (Period 1), two previous (Period 2), and three previous (Period 3) days when using quadratic and cubic polynomial functions.

Figure 1 shows the average milk yield response to increasing thermal load obtained from fitting models M_FR_. For both maximum (Tmax) and average (Tavg) daily temperatures, milk production remained stable within a thermoneutral plateau, followed by a decline beyond the identified HT threshold, indicating a negative impact of heat on milk synthesis. In contrast, the pattern for minimum daily temperature (Tmin) showed a mild upward trend (except for period 0) across the temperature range, suggesting that warmer night-time conditions may coincide with favorable seasonal factors rather than represent true physiological benefits of heat.

Quadratic and cubic polynomials provided similar patterns of response for period 0 and for Tmin in all periods, but differed for Tmax and Tavg for periods involving days previous to the milk recording day. For these cases, the observed HT threshold was higher than for the day of milk recording. The low number of available records, especially at extreme values of the thermal range, can make higher-degree polynomials more sensitive to outliers, which would affect the adjustment of the change points. In this specific case, given the low number of records in this study, quadratic polynomials, which are less sensitive to out of range milk production values, seem more appropriate.

Estimated change points and slopes of change before, between and after the change points for the average population curves fitted with models M_FR_ are shown in Table 2. Change points associated with HT thresholds were those followed by negative slopes of change, indicating a decay in production beyond that temperature. When two HT thresholds were identified, the first one was associated with milder HT smaller decay in production) and the second with more intense HT (larger decay in production). For Tmax, mild and intense HT thresholds were between 20–23 °C and 25–27 °C, respectively, across models. For Tmin, only the response curve for temperature on the day of milk recording identified HT at values of 6 and 12 °C for mild or more intense HT. For Tavg, estimated mild and intense HT were between 11–13 °C and 16–19 °C depending on the polynomial degree and lag period.

Milk loss associated with HT (negative slopes after a change point) also differed for the temperature type, lag period and polynomial degree. For the mild HT, decays were around 8 g/°C for the quadratic polynomials and up to 20–30 g/°C for the cubic polynomials. For intense HT, losses were estimated between 22 and 59 g/°C for the quadratic and between 32 and 85 g/°C for the cubic polynomials.

**Figure 2:**
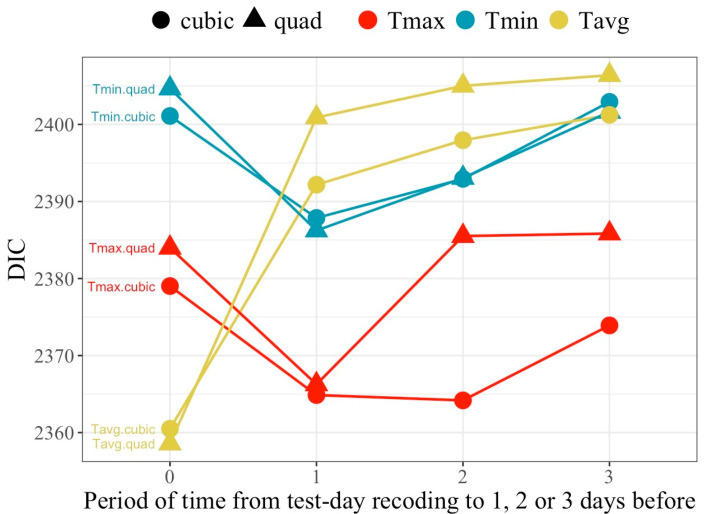
Values of the deviance information criteria (DIC) associated with models fitting either quadratic or cubic polynomial regression of milk yield on temperature measured as the daily maximum (Tmax), minimum (Tmin) or average (Tavg) observed on the day of milk recording (Period 0) or averaged over the day of recording and the previous day (Period 1), or including the previous 2 (Period 2) or 3 (Period 3) days.

Figure 2 presents the values for the goodness of fit criteria, the DIC, for all models regarding average population response (models M_FR_). All models including temperature as a predictor of milk yield exhibited superior goodness of fit relative to models that excluded thermal load effects (DIC = 2410), indicating that heat load measured from temperatures is an influential factor for milk production in this population.

Cubic polynomials exhibited better goodness of fit than the corresponding quadratic fits, except for Tavg on the day of recording, although differences were small in all other cases. Regarding temperature type, model ranking for DIC changed over the lag periods. Tmax and Tmin showed better goodness of fit when accounting for lag effects (periods 1, 2 or 3), while Tavg behaved best for period 0 (no lag effect). Overall, the best fitting model was the one relying on Tavg on the day of recording, closely followed by the models fitting Tmax averaged over periods 1 and 2 for the cubic fit.

### 3.2. Individual Response

**Figure 3:**
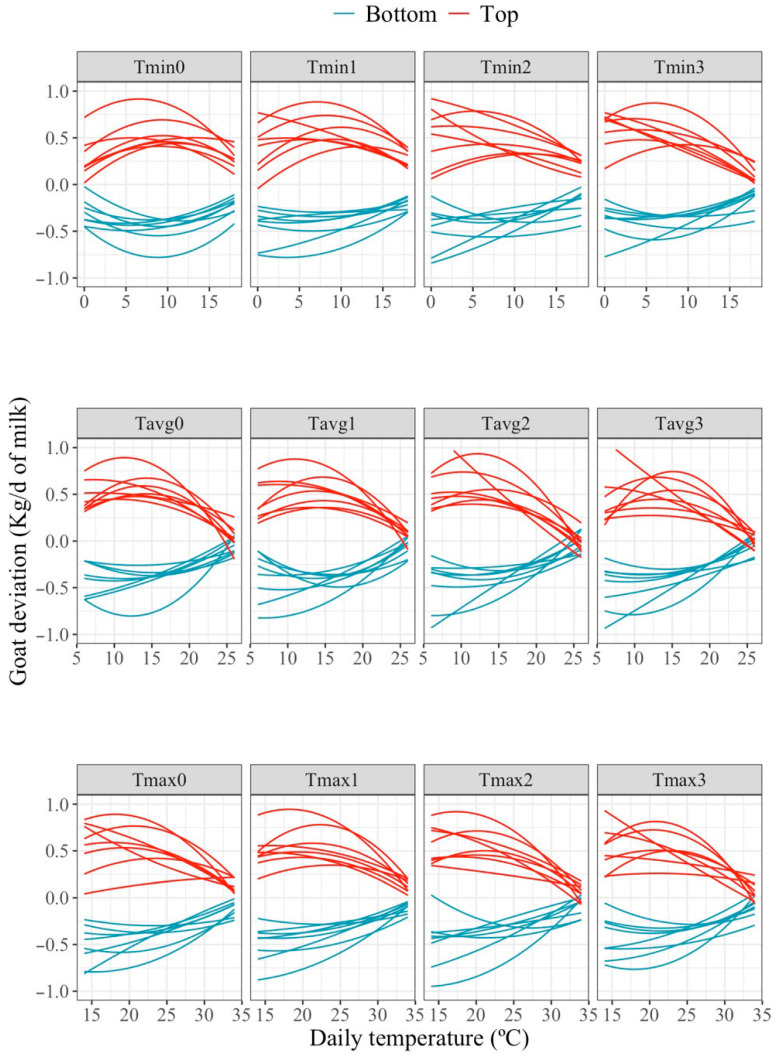
Predicted individual goat deviations in milk production in response to changes in average maximum (Tmax), minimum (Tmin) and mean (Tavg) temperatures recorded during the periods of 1, 2, and 3 days prior to milking for the eight highest-(Top) and eight lowest-(Bottom) goats, classified based on their estimated intercept values.

Figure 3 presents the predicted individual goat deviations in milk production with respect to the average response to changes in minimum (Tmin), maximum (Tmax), and average (Tavg) temperatures recorded at 0 day and in the period of 1, 2, and 3 days before milking for two groups of eight goats classified as the best (Top) and worst (Bottom) goats based on their milk yield at the average heat load (intercept). Please note that curves in this Figure represent the deviation in production for a given animal with respect to the average population response curve, implying that the expected production for that animal at a given temperature would be the sum of the average production estimated for the population and the animal’s individual deviation at that temperature. In other words, animals showing positive/negative individual milk deviation values at a given temperature are expected to produce more/less than the average. Thus, animals showing positive/negative slopes of change in the HT region can be regarded as more/less heat tolerant than the average. Although a variety of individual response patterns can be observed across animals, the EBV for milk production along the studied temperature range tended to decline at high temperatures for the top-ranked animals and vice versa. The pattern was consistent across all temperature variables and time periods evaluated.

A quantification of the distribution of individual slopes of change in production for all the goats under comfort, mild or intense HT is presented in Table 3. Individual response to heat load may vary not only in slopes of change in production but also in HT thresholds. In order to compare individual slopes of response under HT for most animals, two temperature values where individual slopes were evaluated were defined based on HT threshold values specific to each temperature metric (Tmin, Tmax, and Tavg) and for each considered period, shown in Table 2. Estimates of individual slopes at a given temperature value were expected to be normally distributed around a 0-mean value, but unexpectedly highly negative slopes under HT were observed for some goats, especially for Tmax for all periods and also for Tavg for the mild HT average daily value of 16 °C. This might indicate the existence of animals that seem to be notably less tolerant to heat. On the contrary, extremely tolerant animals (animals with anomalously positive slopes of reaction under HT) were not detected. Reaction to increasing minimum daily temperatures showed the smallest variability, indicating that this criterion would be of less interest to improve overall heat tolerance in this population. Differences in reaction between heat tolerant vs. heat susceptible goats ranged from 30 g/°C for Tmin(0−1) under mild HT (Tmin(0−1) = 10 °C) to 200 g/°C for Tmax(0−1) under moderate HT (Tmax(0−1)= 28 °C).

### 3.3. (Co)Variability for Individual Milk and Slope of Milk Loss Under HT

**Figure 4:**
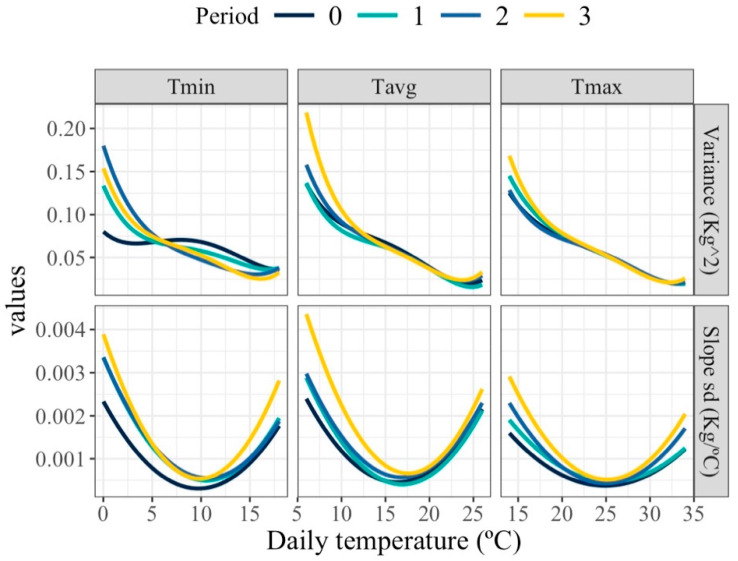
Estimated variance for milk (top row) and standard deviation of slopes of change in milk (bottom row) along the temperature scale under models fitting individual quadratic Legendre polynomials on the average of minimum (Tmin), maximum (Tmax), and average (Tavg) daily temperatures during (0), 1, 2 and 3 days prior to the date of milking.

Figure 4 shows the estimated changes in milk production variance and slopes standard deviation along the temperature scale. Variability in milk yield decreased as temperatures increased at higher temperature ranges. These patterns highlight the complex and dynamic nature of thermal effects on individual milk production variance. The variability in the slope of the response curves was notably greater at both low and high temperature extremes compared to intermediate temperatures. Minimal variability within the thermoneutral zone was anticipated, as milk production is expected to remain relatively stable with increasing temperature in this zone, resulting in slopes approximating zero. These patterns remained consistent across all periods (1, 2, and 3 days before milking date), although subtle differences were observed in the exact profile and steepness of the response curves.

**Figure 5:**
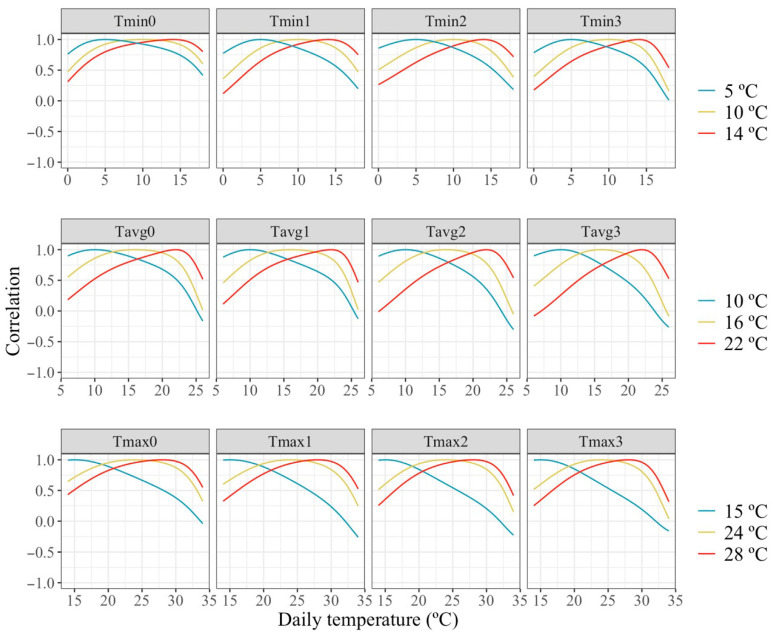
Estimated correlations of change in milk along the temperature scale under models fitting individual quadratic Legendre polynomials on the average of minimum (Tmin), maximum (Tmax), and average (Tavg) daily temperatures during (0), 1, 2 and 3 days prior to the date of milking.

Figure 5 presents the estimated correlations between individual milk production across the temperatures scale and milk production under a temperature representing thermoneutrality and two temperatures representing mild and moderate HT. Estimated correlations were high between similar temperatures but declined rapidly as the temperature increased, reaching values around 0 or slightly negative, with similar patterns across all temperature types and periods. The small estimated correlations between milk production obtained in days with largely different temperatures indicates a marked re-ranking of animals under differing thermal conditions.

**Figure 6:**
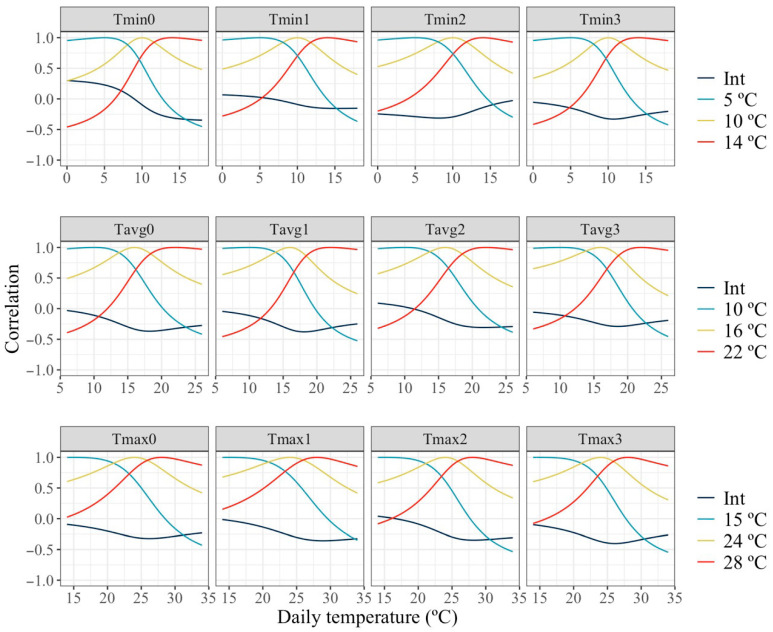
Estimated slopes correlations of change in milk along the temperature scale under models fitting individual quadratic Legendre polynomials on the average of minimum (Tmin), maximum (Tmax), and average (Tavg) daily temperatures during (0), 1, 2 and 3 days prior to the date of milking.

The dynamics of the estimated correlations between baseline milk yield (intercept) and thermal sensitivity (slope traits) across the temperature spectrum is shown in Figure 6. Estimated correlations between the intercept and slopes represent the relationship between the milk production level of the animal and the rate of response in milk to an increase in temperature, which represents thermotolerance under thermal stress. The patterns observed for estimates of this correlation were similar across all cases, showing a decrease from near null values at the lowest temperatures to moderately negative values at higher temperatures. Thus, no relationship between milk production level and rate of change in production under milder temperatures and a small antagonism between high production and thermotolerance under high temperatures were found. Similarly to correlations between levels of production in Figure 5, correlations between slopes under thermoneutrality and two levels of HT with the slopes across all the temperature spectrum are shown. The estimated correlations for slopes of change in production with temperature showed similar patterns to those observed for levels of production in Figure 5. High correlations were found when temperatures for the slopes under comparison are similar and close to null or negative values for slopes at largely dissimilar temperatures.

## 4. Discussion

The present study evaluated the effects of HT on milk production in local Tunisian goats throughout the lactation period, considering milk production as a critical economic trait. Several heat load parameters, including daily minimum, average, and maximum temperatures with consideration of lag effects for the period from the day of milk recording up to 3 days before, were analyzed to identify the most effective thermal load indicator. A limitation of the present study is the unavailability of relative humidity data, which did not allow the computation of the temperature–humidity index (THI), the most widely used integrative indicator of heat load in ruminants). Consequently, the heat-stress thresholds and the estimated milk-yield reductions reported here rely exclusively on temperature-based variables. While this approach is justified by the consistently low humidity conditions characteristic of the hyper-arid environment of southern Tunisia, it may limit the extrapolation of our findings to more humid production systems. Future investigations incorporating THI, should historical humidity records become accessible, would enable a more physiologically robust characterization of heat stress and strengthen the predictive capacity of the proposed models. Comparison of the slopes of milk yield decline after the two HT thresholds (Table 2) indicates that Tmin elicited the smallest response to heat, whereas Tmax and Tavg produced larger declines. Daily maximum temperatures exhibited the lowest variability (CV = 20.0–22.6%), whereas minimum temperatures were the most variable (CV = 45.6–53.1%), with average daily temperatures showing intermediate variability (CV = 25.3–28.2%).

Average daily temperature showed better goodness of fit for the day of recording and larger slopes of decay than maximum or minimum temperature, indicating that it might be a better indicator to detect HT than maximum values. This might be due to the fact that the Tavg is related to minimum as well as maximum temperatures and accounts for the fact that cool night temperatures may counteract the effect of high values during the day [11].

Quantification of HT thresholds and milk production changes in response to increasing heat load has yielded a wide range of estimates in the literature, mainly in cattle and, to less extent in sheep and goats. In the present study, HT thresholds were lower than in other populations, with values of 20/6/13 °C (THI = 64/49/57 with a 30% of relative humidity) for Tmax/Tmin/Tavg for the first change point for mild stress and of 26/11/18 °C (THI = 71/55/62 with a 30% of relative humidity) for Tmax/Tmin/Tavg for the second change point for more severe HT. Most studies find THI values above 77 for HT thresholds for goats (see, e.g., [12]. Slopes of milk yield declines were around 40–50 g/°C, corresponding to approximately 4–5% loss per degree relative to the average daily milk production (~1 kg/day). This reduction is higher than those reported for other dairy populations. For instance, Holstein cows typically show declines of 1–2% per °C above the thermoneutral zone [3,9,13,14,15], Mediterranean dairy sheep exhibit moderate decreases depending on lactation stage [16], and selected dairy goats show smaller relative losses under similar HT conditions [5]. Both results estimated HT thresholds and slopes of milk decay under HT point at a relatively high impact of HT observed in this local Tunisian goat population. Several hypotheses might explain this unexpected result. First, as suggested by Berman [17], animals adapted to harsh environments often prioritize survival over production, which may lead to reduced milk yield even before direct heat effects occur. Associated with this hypothesis, as opposed to highly productive breeds, which tend to prioritize energy allocation to milk production at the expense of body reserves and functional needs [18]. This breed has not been traditionally used for milking purposes. Then, even under relatively favorable food conditions, such as the supplementation with concentrate to enhance production, a prioritization to accumulate body reserves over maintaining milk production might be expected [19]. The lack of selection for milk production, together with the HT effects might then result in a larger than expected impact of high temperatures in milk production. Another possible explanation is that the stage of lactation, although included in the models of analyses may be a confounding factor. Higher temperatures coincided with periods of declining production, potentially amplifying the observed effects. Finally, semi-intensive management with variable feed availability and natural pasture quality may exacerbate heat effects compared with intensively managed dairy herds. We acknowledge that adding information from physiological indicators of heat, increased respiration rates or rectal temperatures, decreased feed intake or increased water consumption beyond the detected thresholds might be needed to confirm our findings for HT effect using changes in observed milk production under changing temperatures. Moreover, we recognize that the doubling morning milking yields to estimate daily production may overestimate milk production under HT given that afternoon/evening yield may be lower due to more severe HT effects during that part of the day. Nevertheless, we believe that this would not largely modify the estimated biological pattern of declining milk production under the highest temperatures of the studied period.

In this study, the variability of individual milk yield decreased as temperature increased, and showed a slight trend to increase at the highest values of temperature. Typically, in unfavorable environments, a reduction in variability of productive traits can occur as animals are constrained in expressing their full production potential. Then, at extremely high temperatures, variability might be expected to increase again due to large variability of slopes of response in this region. However, we did not include results for these very high temperatures because available data were scarce.

The results from the literature regarding the effect of HT on milk production variability differ across studies. Hammami et al. [20] reported a reduced variability of milk yield under heat, suggesting that unfavorable thermal conditions may limit the expression of genetic potential. Conversely, Ravagnolo and Misztal [21] observed higher variability for milk yield in Holstein cows under elevated temperature–humidity index (THI), while Bernabucci et al. [14] also reported greater variability in milk traits during hot periods. Similarly, Cheruiyot et al. [22] found that genetic variation in milk yield increased with THI, indicating that individual differences in heat tolerance play a significant role in production responses.

Considerable individual variation was observed in milk responses to changes in temperature in local Tunisian goats. Differences in the slopes of milk yield change indicate that some animals are more sensitive to increasing temperatures than others, highlighting variation in individual adaptability within the population, agreeing with findings by Bohmanova et al. [23]. Variability in individual response to increasing temperatures provides expectation for genetic selection response. However, the relatively lower variability in slopes, compared with that in milk level, further suggests a less effective selection potential on heat tolerance than for milk level.

## 5. Conclusions

In this study, we evaluated the impact of HT on milk production in local goats throughout lactation. Our study shows that high temperatures reduce goat performance, with clear decreases in milk yield. We observed individual animal variability in both, milk yield and its changes under high temperatures, which offers a useful basis for genetic selection for heat tolerance by using the slope of decline in production as a breeding tool. Moreover, goats with higher milk production tended to decrease their production under heat. This indicates an antagonistic relationship between increased milk yield and heat tolerance, which should be taken into account when designing the breeding strategy.

## Figures and Tables

**Table 1:** Summary statistics (Minimum (Min), 25th percentile (P25), 75th percentile (P75), Mean, Standard Deviation (SD), and Maximum (Max)) for the number of milk records per goat (N), days in milk (DM), milk production (MP, kg), and daily temperature (°C) indices (Tmin, Tmax, Tavg) recorded on the day of milking (day 0) and averaged over days 0 and days 1, 2, and 3 before the recording date.

Variables	Min	P25	Mean	SD	P75	P90	Max
N	2	7.25	11		22		44
DM	7	46	71	36.77	99		180
MP	0.20	1.00	1.20	0.55	1.60		5.20
Tmax_0_	14.16	20.13	22.45	5.08	26.56	30.78	43.60
Tmax(0−1)	14.12	20.19	22.57	4.84	25.82	29.86	43.60
Tmax(0−2)	14.46	20.05	22.95	4.60	26.35	29.49	41.89
Tmax(0−3)	14.88	19.80	22.55	4.56	26.28	28.70	40.45
Tmin_0_	0.03	4.94	8.70	4.62	12.20	15.45	24.74
Tmin(0−1)	0.67	6.02	8.56	3.91	10.99	13.66	24.74
Tmin(0−2)	0.83	6.39	7.80	3.81	9.92	14.01	23.76
Tmin(0−3)	1.28	6.60	8.15	3.72	10.21	13.66	22.96
Tavg_0_	6.50	13.55	14.81	4.17	18.52	21.30	33.29
Tavg(0−1)	7.58	13.05	15.36	4.01	18.42	21.23	33.29
Tavg(0−2)	7.63	13.06	15.33	3.91	18.13	20.69	32.41
Tavg(0−3)	8.04	12.66	15.42	3.90	18.20	21.21	31.81

**Table 2:** Estimated change points (chp1, chp2) for temperatures (°C) and slopes (g/°C) before the first change point (Slp_b1), between change points (Slp_1-2) and after the second change point (Slp_a2) for the quadratic and cubic polynomial curves fitted as response in milk yield to changes in combinations of temperature type (daily maximum, minimum or average) and lag period (involving the day of recording, 0, or the average with previous 1, 2 or 3 days).

	Quadratic Polynomial					Cubic Polynomial				
	Slp_b1	chp1	Slp_1-2	chp2	Slp_a2	Slp_b1	chp1	Slp_1-2	chp2	Slp_a2
Tmax_0_	10.49	20	−8.51	26	−28.95	56.96	18	0.70	22	−33.99
Tmax(0−1)	14.25	20	−13.59	25	−42.60	−1.72	23	−25.77	27	−72.97
Tmax(0−2)	16.08	20	−7.81	25	−34.37	−50.07	17	2.80	26	−85.23
Tmax(0−3)	13.01	21	−11.88	25	−38.67	−29.87	18	12.37	27	−57.69
Tmin_0_	−6.15	6	−9.29	11	−12.25	−29.80	4	−0.57	12	−31.89
Tmin(0−1)	−11.95	6	14.97	10	39.96	11.58	7	33.62	11	65.34
Tmin(0−2)	−17.38	6	10.80	10	36.45	−19.54	9	9.23	11	55.16
Tmin(0−3)	−21.31	7	3.83	11	28.98	−41.48	8	−15.46	11	32.44
Tavg_0_	14.73	13	−21.08	18	−59.45	7.96	13	−31.54	18	−68.46
Tavg(0−1)	15.27	13	−6.37	17	−27.93	−33.11	11	11.08	18	−51.78
Tavg(0−2)	8.44	12	−6.86	16	−22.84	−23.56	11	9.42	18	−45.77
Tavg(0−3)	−5.61	13	−13.67	17	−21.79	−64.94	12	−20.74	19	−59.15

**Table 3:** Quantile distribution for estimated values of individual slopes of change in milk (g/°C) under mild (HT1) and moderate (HT2) from random regression models fitting quadratic Legendre polynomials on the average of minimum (Tmin), maximum (Tmax), and average (Tavg) daily temperatures during four periods, starting at the day of milking and going back 0, 1, 2 or 3 days. Temperatures representing HT1 and HT2 for each temperature type are shown underneath each condition for each temperature type.

		Tmax		Tmin		Tavg	
Period	Quantile	HT1 24 °C	HT2 28 °C	HT1 10 °C	HT2 14 °C	HT1 16 °C	HT2 22 °C
0	0%	−123	−180	−91	−20	−163	−45
	5%	−72	−113	−58	−17	−85	−30
	25%	−17	−9	−13	−10	−21	−9
	75%	1	22	2	11	3	8
	95%	10	39	36	30	11	38
	100%	39	52	66	46	84	60
[0, 1]	0%	−132	−175	−17	−48	−51	−31
	5%	−77	−116	−14	−15	−38	−19
	25%	−21	−16	−8	−7	−19	−5
	75%	3	21	13	9	−4	12
	95%	22	49	34	29	18	48
	100%	40	65	46	69	28	85
[0, 2]	0%	−140	−24	−36	−38	−134	−54
	5%	−79	−15	−20	−20	−73	−24
	25%	−5	−8	−9	−2	−27	−3
	75%	15	5	14	8	0	18
	95%	45	18	39	17	21	48
	100%	66	30	75	32	41	66
[0, 3]	0%	−135	−204	−31	−43	−51	−41
	5%	−82	−125	−22	−20	−42	−29
	25%	−21	−13	−9	−6	−21	−4
	75%	3	4	7	5	−6	18
	95%	21	50	34	31	11	42
	100%	43	71	49	68	25	64

## Data Availability

All datasets generated or analyzed in this research are included in the published article. Additional information can be requested from the corresponding author.
